# Association between endometriosis and risk of systemic lupus erythematosus

**DOI:** 10.1038/s41598-020-79954-z

**Published:** 2021-01-12

**Authors:** Yu-Hsi Fan, Pui-Ying Leong, Jeng-Yuan Chiou, Yu-Hsun Wang, Ming-Hsiang Ku, James Cheng-Chung Wei

**Affiliations:** 1 School of Medicine, Chung Shan Medical University, Taichung City, 40201 Taiwan; 2 Institute of Medicine, Chung Shan Medical University, Taichung City, 40201 Taiwan; 3 Division of Allergy, Immunology and Rheumatology, Department of Medicine, Chung Shan Medical University Hospital, Taichung City, 40201 Taiwan; 4 School of Health Policy and Management, Chung Shan Medical University, Taichung City, 40201 Taiwan; 5 Department of Medical Research, Chung Shan Medical University Hospital, Taichung City, 40201 Taiwan; 6 Department of Family and Community Medicine, Chung Shan Medical University Hospital, Taichung City, 40201 Taiwan; 7 Graduate Institute of Integrated Medicine, China Medical University, Taichung City, 40201 Taiwan

**Keywords:** Diseases, Medical research, Rheumatology, Risk factors

## Abstract

To examine the association between endometriosis and the risk of systemic lupus erythematosus (SLE), this nationwide, population-based, retrospective cohort study was conducted based on National Health Insurance Research Database in Taiwan. Endometriosis (N = 16,758) and non-endometriosis (N = 16,758) groups were identified by matching baseline characteristics and comorbidities. Student’s t-tests and the Kaplan–Meier estimator were utilized to estimate the hazard ratio (HR) and cumulative probability of SLE in the two groups. The endometriosis group showed a significantly higher incidence density rate (0.3 vs. 0.1 per 1000 person-years) and hazard ratio in SLE group (adjusted HR [aHR], 2.37; 95% confidence interval [CI] 1.35–4.14) compared to the non-endometriosis group. Subgroup analysis revealed that patients with endometriosis between 30 and 45 years of age, or were non-steroidal anti-inflammatory drug users, or were hormonal medications-free participants, had higher risks of SLE. For patients with endometriosis, surgical intervention did not significantly impact on the risk of SLE. Our results demonstrated an increased risk of SLE in patients with endometriosis. Clinicians should be aware of this association when managing patients with endometriosis or SLE.

## Introduction

Endometriosis is one of the most common gynaecological disorders that affects nearly 10% of menstruating women^1^. Pelvic pain and infertility are common symptoms of endometriosis^2^. Although the causes of endometriosis remain uncertain, one hypothesis is retrograde menstruation. It is assumed that, as a result of retrograde menstruation, endometrial tissues adhere to pelvic organs and peritoneal surfaces, which subsequently become ectopic endometrial tissues. Ectopic endometrial tissues, just like the endometrium, proliferate and shed as they are regulated by the level of oestrogen and progesterone. The exfoliating tissues stimulate the inflammatory response and result in adhesion of fibrous tissue and endometrial tissue, which may eventually precipitate into chronic inflammation and infertility^3,4^.

Evidence has shown that that the pathogenesis of endometriosis is involved in immune system dysfunction. Specific anti-endometrium antibodies were found in patients with endometriosis, which can explain the pain and chronic inflammation^5^. Changes in cytokine concentration were also detected in peritoneal and follicular fluids^6^. Anti-nuclear antibodies (ANA) were detected in 18% of patients with endometriosis, while no patients expressed ANA in the control group^7–9^.

Systemic lupus erythematosus (SLE) is an autoimmune disease that affects multiple organs. The prevalence of SLE is higher in females than males at a 1:9 ratio. Its incidence is highest in females of early reproductive age, which is similar to endometriosis.

Recent studies have revealed a higher prevalence of allergies and autoimmune diseases in patients with endometriosis^10–12^. However, the relationship between endometriosis and SLE has not been studied thoroughly and remains unclear. Owing to limited number of cohort studies focusing on this topic, we aimed to investigate the association between endometriosis and the risk of SLE using data from Taiwan’s National Health Insurance Research Database (NHIRD) in this population-based retrospective cohort study.

## Methods

### Data source

The Taiwanese government established Taiwan’s National Health Insurance (NHI) program in 1995, which is a nationwide, government-run compulsory insurance program, to provide universal health coverage to citizens and foreigners in Taiwan. In 2014, the program covered over 99.9% of Taiwan’s population^13^. Registration files and claimed data including registry of patient's diagnosis, drug prescriptions and received medical service, etc., from the NHI program were collected and sorted into the National Health Insurance Research Database (NHIRD) through full encryption. The Longitudinal Health Insurance Database (LHID) served as the dataset for this cohort, which yielded a sample of 1 million participants from 23 million beneficiaries of the NHIRD.

As all personal data in this database had been multiply encrypted, the informed consent was waived by the approving committee. This study complied with relevant laws and regulations, and it was approved by the Chung Shan Medical University Hospital Institutional Review Board (CS15134).

### Study group and outcome measurement

This research adopted a retrospective cohort study design. First, female subjects were selected from the one million participants acquired from the LHID. Next, we identified the endometriosis group with patients with newly diagnosed endometriosis (The International Classification of Diseases, 9th Revision, Clinical Modification [ICD-9-CM] codes = 617) from 2000 to 2011 confirmed with gynaecological ultrasonography or laparoscopy. The non-endometriosis group consisted of female participants who were never diagnosed with endometriosis from 1999 to 2013. In addition, any subject diagnosed with SLE before the index date was also excluded.

The index date of this cohort was the date of the first endometriosis diagnosis for a patient. The outcome measurement was the diagnosis of SLE (ICD-9-CM = 710.0) and usage of hydroxychloroquine for one year. The end point of this cohort was the occurrence of SLE (December 31st, 2013) or withdrawal from the NHI program, whichever occurred first.

### Covariates and matching

Age, comorbidities, use of corticosteroids, use of NSAIDs and use of hormonal medications were selected as potential confounders. Previous studies had shown the potential association between these variables and SLE^14–17^. Their associations with endometriosis were demonstrated in the present study (Table 1). We used the ICD-9-CM codes for identification of the comorbidities of this cohort including hypertension (ICD-9-CM codes = 401–405), hyperlipidaemia (ICD-9-CM codes = 272.0–272.4), chronic liver disease (ICD-9-CM code = 571), major depressive disorder (ICD-9-CM codes = 2962, 2963), chronic obstructive pulmonary disease (ICD-9-CM codes = 490–492, 494, 496), diabetes (ICD-9-CM code = 250), coronary artery disease (ICD-9-CM codes = 410–414), and cerebrovascular disease (ICD-9-CM codes = 430–438). Data on the use of medications mentioned in this study was available from LHID, which included any prescriptions of prescription drugs. Credible diagnoses of endometriosis were defined as an assessment using gynaecological ultrasonography or laparoscopy. Corticosteroids or NSAIDs or hormonal medications use were defined as use for more than 30 days during the observation period. Surgical treatment for endometriosis included laparoscopic surgery, adnexectomy, and hysterectomy. The entire list of surgeries as surgical treatment for endometriosis can be found in Supplementary Table S1.

**Table 1:** Demographic characteristics of the endometriosis and non-endometriosis groups.

Characteristic	Before PSM				*P* value	After PSM				*P* value
	Endometriosis (N = 16,578)		Non-endometriosis (N = 100,548)			Endometriosis (N = 14,967)		Non-endometriosis (N = 29,934)		
	n	%	n	%		n	%	n	%	
**Age**					1					0.117
< 30	3645	21.8	21,870	21.8		3645	21.8	3645	21.8	
30–45	8935	53.3	53,610	53.3		8935	53.3	8778	52.4	
≥ 45	4178	24.9	25,068	24.9		4178	24.9	4335	25.9	
Mean ± SD	38 ± 9.4		38 ± 9.4		1	38 ± 9.4		38 ± 9.4		0.676
**Comorbidities**										
Hypertension	746	4.5	3598	3.6	** < 0.001**	746	4.5	744	4.4	0.958
Hyperlipidemia	316	1.9	1451	1.4	** < 0.001**	316	1.9	310	1.8	0.809
Chronic liver disease	308	1.8	1149	1.1	** < 0.001**	308	1.8	311	1.9	0.903
Major depressive disorder	116	0.7	474	0.5	** < 0.001**	116	0.7	114	0.7	0.895
COPD	163	1.0	629	0.6	** < 0.001**	163	1.0	159	0.9	0.823
**Diabetes**	305	1.8	1660	1.7	**0.114**	305	1.8	295	1.8	0.680
Coronary artery disease	136	0.8	585	0.6	** < 0.001**	136	0.8	128	0.8	0.621
Cerebrovascular disease	62	0.4	308	0.3	**0.174**	62	0.4	58	0.3	0.715
Corticosteroids	8881	53.0	38,684	38.5	** < 0.001**	8881	53.0	8881	53.0	1.000
NSAIDs	11,953	71.3	53,653	53.4	** < 0.001**	11,953	71.3	11,961	71.4	0.923
Hormonal medications	5292	31.6	11,013	11.0	** < 0.001**	5292	31.6	5289	31.6	0.972

Bold font represents statistical significance (*P* < 0.05). Propensity score matching (PSM) by age, comorbidities, corticosteroids use, NSAIDs use, and hormonal medications use.

*SLE* Systemic lupus erythematosus, *PSM* propensity score matching, *COPD* chronic obstructive pulmonary disease, *NSAIDs* non-steroidal anti-inflammatory drugs.

**Figure 1:**
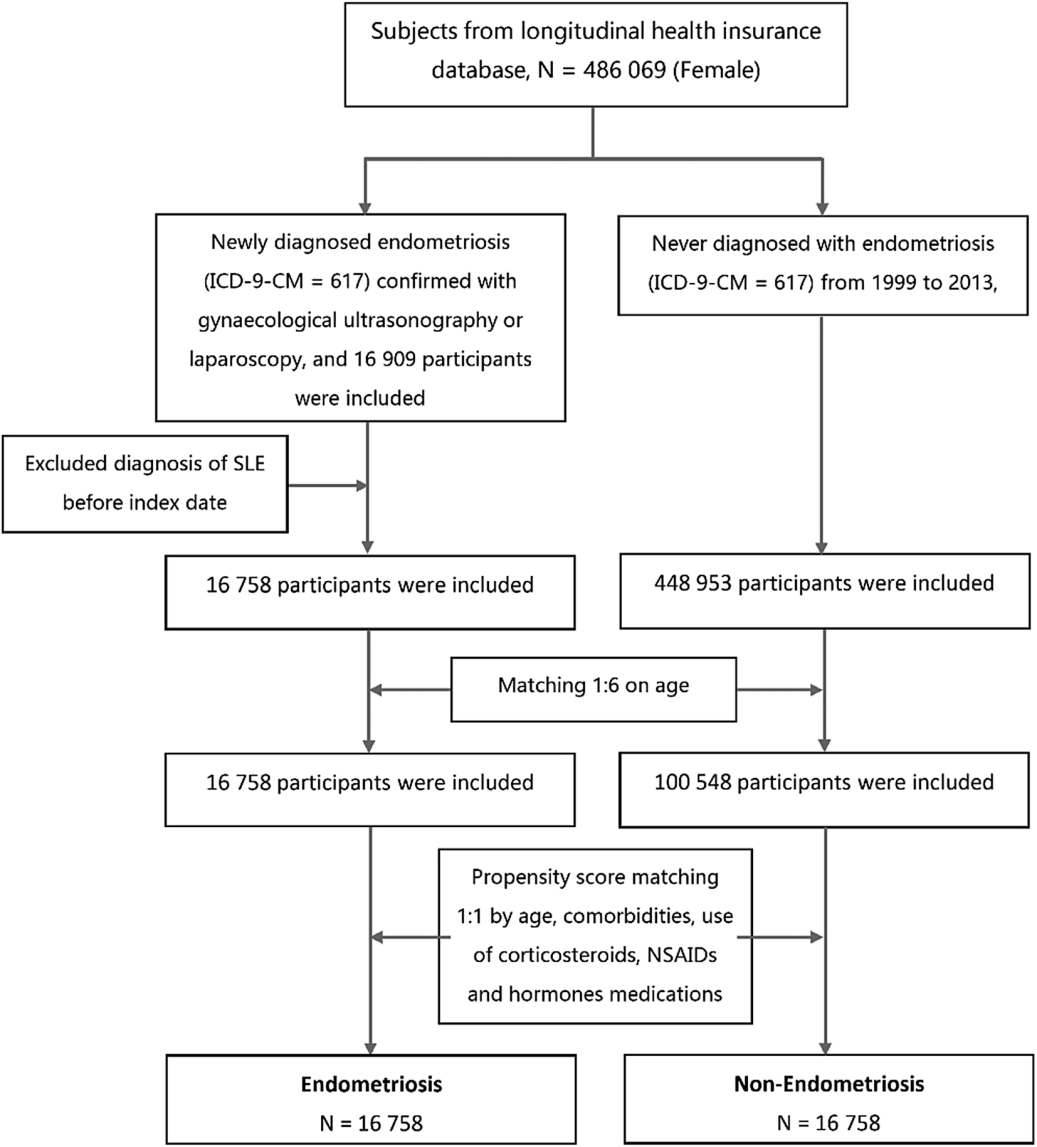
Description of study design and identification of this cohort study.

First, we used a 1:6 age matching for the endometriosis (N = 16,758) and non-endometriosis groups (N = 100,548). Propensity score matching was performed by accounting for covariates including age, comorbidities, use of corticosteroids, use of NSAIDs, and use of hormonal medications to control confounding between the endometriosis and non-endometriosis groups. Finally, the endometriosis group (N = 16,785) and non-endometriosis group (N = 16,785) were identified after matching (Fig. 1).

### Statistical analysis

To compare the baseline characteristics of both groups, a chi-squared test and independent t-test were utilized respectively for categorical variables and continuous variables. The cumulative probability of SLE in the two groups was estimated using the Kaplan–Meier estimator. A log-rank test was then applied to test for statistical significance. The Cox proportional-hazards model calculated HR and aHR of SLE for the variables, and 95% CI showed the magnitude and range of them. We further conducted a sensitivity analysis, limiting the endometriosis diagnostic criteria to laparoscopic-diagnosed endometriosis to assess the robustness of the results. In subgroup analysis, we separated the participants into subgroups by different variables, including age, use of corticosteroids, use of NSAIDs, use of hormonal medications, and surgical treatment to clarify their effects on the association between endometriosis and SLE. Further, interaction analysis was performed to compare the risks of SLE between different subgroups. A two-tailed *P* value of < 0.05 was considered statistically significant. Statistical analyses were performed using SPSS 18.0 (Version 18.0, SPSS Inc., Chicago, USA).

## Results

Before matching, for the endometriosis group (N = 16,578), the number of female patients with hypertension, hyperlipidaemia, chronic liver disease, major depressive disorder, chronic obstructive pulmonary disease, coronary artery disease, corticosteroids use, NSAIDs use, and hormonal medications use was significantly greater than the number of females with these conditions/criteria in the non-endometriosis group (N = 100,548; *P* < 0.05; Table 1). After propensity score matching, there was no significant difference in the baseline factors between the two groups (*P* > 0.05).

In Table 2, the number of SLE cases and person-years, the incidence density rate (per 1000 person-years), and crude/adjusted HR were calculated for each category using the Cox proportional-hazards model. In the non-endometriosis group, 18 new cases of SLE occurred during 126,860 person-years, while there were 39 cases of new-onset SLE during 113,985 person-years in the endometriosis group. Difference in the incidence density rate of SLE was significantly different between the two groups after adjusting for confounders, including age, gender, comorbidities, and drug use. The endometriosis group showed a significantly higher incidence density rate (0.3 vs. 0.1) and HR/aHR compared with the non-endometriosis group (aHR, 2.37; 95% CI 1.35–4.14). No significant effects of age, hypertension, chronic liver disease, the uses of corticosteroids, NSAIDs, and hormonal medications were observed in the HR/aHR of SLE with the Cox proportional-hazards model. To evaluate the reliability of the association, we carried out a sensitivity analysis by limiting the endometriosis diagnostic criteria to laparoscopic-diagnosed endometriosis, which is in line with the current gold standard of endometriosis diagnosis (Supplementary Table S2). Similar association was demonstrated in the sensitivity analysis. Comparing with the non-endometriosis group, a significant higher aHR for SLE was demonstrated in patients with laparoscopic-diagnosed endometriosis (aHR, 4.74; 95% CI 1.07–20.93).

**Table 2:** Cox proportional-hazards model analyses HRs and 95% CIs of SLE of each variables.

Characteristics	Number of SLE cases	Person-years	Incidence density rate	Crude HR	95% CI	Adjusted HR	95% CI
**Endometriosis**							
No	18	126,860	0.1	1		1	
Yes	39	113,985	0.3	**2.36**	**1.35–4.13**	**2.37**	**1.35–4.14**
**Age (years)**							
< 30	13	55,816	0.2	1			1
30–45	47	196,087	0.2	1.00	0.57–1.73	0.99	0.57–1.73
≥ 45	23	79,681	0.3	1.19	0.64–2.23	1.22	0.64–2.31
**Comorbidities**							
Hypertension	2	9307	0.2	0.87	0.21–3.57	0.82	0.19–3.46
Chronic liver disease	2	4559	0.4	1.90	0.46–7.77	1.94	0.47–8.02
Corticosteroids	38	148,859	0.3	1.34	0.77–2.33	1.72	0.94–3.15
NSAIDs	40	193,804	0.2	0.63	0.35–1.11	0.54	0.29–1.01
Hormonal medications	15	89,857	0.2	0.63	0.35–1.14	0.63	0.34–1.15

Bold font represents statistical significance (*P* < 0.05). Incidence density rate is defined per 1000 person-years. An adjusted hazard ratio (HR) is calculated from crude HR after adjusting for age, hypertension, chronic liver disease, corticosteroids use, NSAIDs use, and hormonal medications use. Hyperlipidemia, major depressive disorder, chronic obstructive pulmonary disease, diabetes, coronary artery disease, and cerebrovascular disease were eliminated in the model since only one or no SLE cases were observed.

*SLE* systemic lupus erythematosus, *HR* hazard ratio, *NSAIDs* non-steroidal anti-inflammatory drugs, *CI* confidence interval.

**Figure 2:**
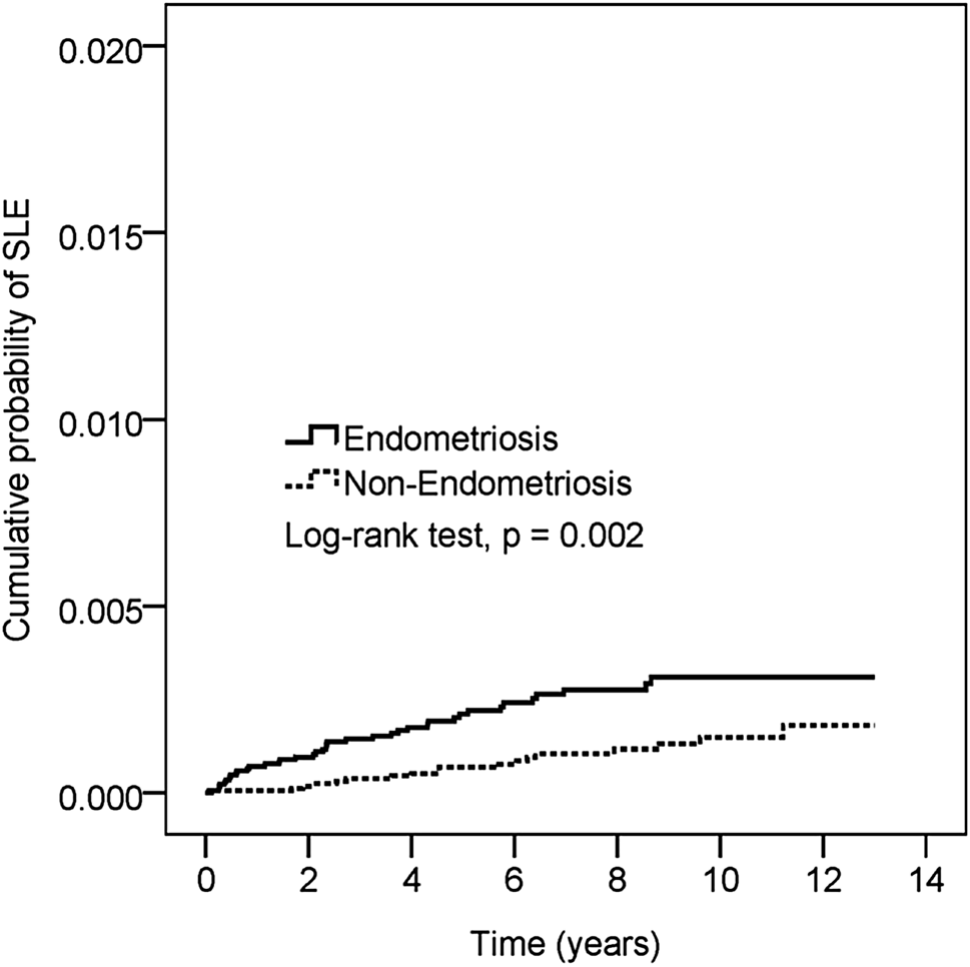
Kaplan–Meier estimator and the log-rank test for cumulative probability of endometriosis and non-endometriosis group.

To estimate the difference in the cumulative probability of SLE between the two groups, we used the Kaplan–Meier estimator and log-rank test to conduct further examination (Fig. 2). In Fig. 2, the endometriosis group had a significantly higher cumulative probability of new-onset SLE during the study period (*P* = 0.002).

Table 3 demonstrates patients with new-onset SLE in both groups were further analysed by using diverse variables (different ages, with or without the use of corticosteroids, NSAIDs or hormonal medications). Interaction analysis was utilized to compare the risk of SLE across different subgroups. For those who were between 30 and 45 years of age, patients with endometriosis had an increased risk of SLE (HR, 3.33; 95% CI 1.42–7.81). There were no significant differences in SLE risks between participants less than 30 years of age and greater than or equal to 45 years of age (HR, 1.76; 95% CI 0.57–5.38 and HR, 1.73; 95% CI 0.61–4.86, respectively; *P* values for interaction = 0.521). Corticosteroids-free participants (who had never used corticosteroids or used corticosteroids for < 30 days during follow up) who had endometriosis were observed for a higher hazard of SLE (HR, 9.29; 95% CI 2.14–40.25). On the other hand, among corticosteroids users (who had used corticosteroids for ≥ 30 days during follow up), patients with endometriosis were not observed for a higher risk of SLE (HR, 1.45; 95% CI 0.76–2.77; P values for interaction = 0.019). In contrast, a significantly increased risk of SLE for patients with endometriosis was displayed in NSAIDs users (who had used NSAIDs for ≥ 30 days during follow up; HR, 2.51; 95% CI 1.28–4.94), but not in NSAID-free participants (who had never used NSAIDs or used NSAIDs for < 30 days during follow up; HR, 2.09; 95% CI 0.77–5.66; P values for interaction = 0.804). As for hormonal medications, a significant higher risk of SLE was observed in the group of hormonal medication-free participants (who had never used hormonal medications or used hormonal medications for < 30 days during follow up; HR, 2.17; 95% CI 1.14–4.13), but not for hormonal medications users (who had used hormonal medications for ≥ 30 days during follow up; HR, 3.03; 95% CI 0.97–9.53; P values for interaction = 0.651).

**Table 3:** Subgroup analysis for HRs and 95% CIs of SLE according to different variables.

Characteristics	Endometriosis		Non-endometriosis		HR	95% CI
	n	Number of SLE cases	n	Number of SLE cases		
**Age (years)**						
< 30	3645	8	3645	5	1.76	0.57–5.38
30–45	8935	22	8778	7	**3.33**	**1.42–7.81**
≥ 45	4178	9	4335	6	1.73	0.61–4.86
P for interaction = 0.521						
**Corticosteroids**						
No	7877	17	7877	2	**9.29**	**2.14–40.25**
Yes	8881	22	8881	16	1.45	0.76–2.77
P for interaction = 0.019						
**NSAIDs**						
No	4805	11	4797	6	2.09	0.77–5.66
Yes	11,953	28	11,961	12	**2.51**	**1.28–4.94**
P for interaction = 0.804						
**Hormonal medications**						
No	11,466	28	11,469	14	**2.17**	**1.14–4.13**
Yes	5292	11	5289	4	3.03	0.97–9.53
P for interaction = 0.651						

Bold font represents statistical significance (*P* < 0.05). Hazard ratio (HR) was estimated using the univariate Cox proportional-hazards model.

*SLE* Systemic lupus erythematosus, *HR* hazard ratio, *NSAIDs* non-steroidal anti-inflammatory drugs.

In Table 4, the endometriosis group was divided into two subgroups, the surgical treatment group (those who had received surgical intervention including laparoscopic surgery, adnexectomy, or hysterectomy) and the non-surgical treatment group (those who did not receive any surgical intervention), to investigate whether surgical interventions affected the risk of SLE in patients with endometriosis. When being compared with the non-endometriosis group, both surgical treatment endometriosis group and non-surgical treatment endometriosis group had a higher risk in SLE (aHR 1.61; 95% CI 0.59–4.36 and aHR 2.55; 95% CI 1.44–4.51, respectively). By further comparing the surgical treatment endometriosis group and non-surgical treatment endometriosis group, no statistically significant difference was found between their risks of SLE (aHR 0.61; 95% CI 0.24–1.58).

**Table 4:** Cox proportional-hazards model analyses HRs and 95% CIs of SLE for patients with endometriosis with surgical treatment.

Characteristics	N	Number of SLE cases	Crude HR	95% CI	Adjusted HR	95% CI
**Endometriosis**						
No	16,758	18	1		1	
Non-surgical treatment	13,678	34	**2.58**	**1.45–4.57**	**2.55**	**1.44–4.51**
Surgical treatment	3080	5	1.51	0.56–4.06	**1.61**	**0.59–4.36**
**Endometriosis alone**						
Non-surgical treatment	13,678	34	1		1	
Surgical treatment	3080	5	0.60	0.23–1.52	0.61	0.24–1.58

Bold font represents statistical significance (*P* < 0.05). Hazard ratio (HR) was estimated using the univariate Cox proportional-hazards model.

*SLE* Systemic lupus erythematosus, *HR* hazard ratio.

## Discussion

The associations between endometriosis and SLE remain obscure due to limited number of relevant cohort studies. One of the earliest research studies was conducted by the Endometriosis Association in 1998. A cross-sectional research study featuring 5500 female patients from the USA showed a prevalence odds ratio of 20.7 (95% CI 14.3–20.9) for SLE^18^. Nonetheless, this study was limited by its highly self-driven participants and the lack of adjustment for confounders in the statistical analysis. An elevated risk of SLE was confirmed by a 22-year follow-up study featuring nurses with 6434 patients laparoscopically diagnosed with endometriosis (HR 2.03; 95% CI 1.17–3.51)^19^. Recently, this correlation was also reported by a case–control study using Swedish registers (odds ratio 1.39; 95% CI 1.09–1.78)^20^. The advantage of this research was its substantial follow-up time, but the lack of matching for specific variables, such as comorbidities, drugs, etc., had become a limitation of this study.

A Danish cohort study did not support the same association as above. This study analysed 9191 patients whose endometriosis were diagnosed by laparoscopy or laparotomy^21^. There were no significant associations between endometriosis and SLE (standardized incidence ratio = 1.1; 95% CI 0.6–2.1). However, a modest increase in the risk of SLE was mentioned in the study.

The present study is one of the largest cohort studies focusing on the association between endometriosis and SLE in patients from the LHID—a nationwide database from Taiwan with over one million participants. This study reports a significant association between endometriosis and SLE (aHR 2.37; 95% CI 1.35–4.14). By limiting the endometriosis diagnostic criteria to laparoscopic-diagnosed endometriosis, this association still remains. In addition, patients with endometriosis who were corticosteroid-free, NSAIDs-using, hormonal medications-free, or between the age of 30 to 45 have a greater risk in developing SLE. Furthermore, for patients with endometriosis, regardless of whether they received surgical treatment, there was no significant difference in the risk of SLE.

Aberration of the immune system could explain the correlation between endometriosis and SLE. Several pieces of evidence imply that an overactive adaptive immune system may play a significant role in the pathogenesis of endometriosis and SLE. ANA was detected in 18% of patients with endometriosis, 100% of patients with SLE, and was not detected in the control group in a prospective randomized trial^7^. In addition, intense B cell activation and tumour necrosis factor-α (TNF-α) upregulation were observed in both the endometriosis and SLE groups^22,23^. Characteristics of underactivity in innate immune cells were also observed in the SLE and endometriosis groups. A recent study demonstrated that neutrophils had a decreased rate of apoptosis in patients with endometriosis compared with disease-free females^24,25^. As for patients with SLE, the dysfunctional phagocytic ability of neutrophils is well-acknowledged and contributes to vascular damage, lupus nephritis, and skin disease in patients with SLE^26,27^. Although it is still unclear how the abnormal expression of the immune system participates in the pathogenesis of endometriosis and SLE, this would be a reasonable explanation for the association between endometriosis and SLE.

The dysfunction of cytokine networks and complementary systems could be another mechanism linking endometriosis and SLE. Changes in cytokine concentrations in peritoneal and follicular fluids were discovered in patients with endometriosis, and a higher level of mannan-binding lectin serine protease 1 (MASP-1) was found both in patients with endometriosis and in patients with SLE^6,28,29^. MASP-1 has various functions, including activation of the complement and cytokine system, and regulation of endothelial cell function^30^. Moreover, a recent animal study evidenced the involvement of MASP-1 in the development of lupus-like glomerulonephritis in mice^31^. However, our current understanding of MASP-1 still cannot prove whether a higher amount of MASP-1 is related to the pathogenesis of endometriosis or SLE.

Hormones, especially oestrogen, had been proven playing an important role in the development of both endometriosis and SLE^2,32^. The incidence of the two diseases increases after menarche and decreases after menopause. Overexpression of oestrogen receptor α (ERα) mRNA was found in endometrial tissue, which is related to the development and growth of endometrial tissue^33^. Recent research has also reported the exaggerated response of patients with SLE to oestrogen^34^. However, it is still largely unknown how oestrogen participates in the emergence of endometriosis and SLE. Our study reported that endometriosis significantly increased the risk of SLE among hormonal medications-free participants. Even higher risk of SLE was observed in hormonal medications users, although there was no statistical significance in this association. Smaller sample size in the subgroup and the excessively broad classification of hormonal medications may be the reason. Because of the scarcity and inconsistency of the results, studies with larger sample sizes and more detailed classification of hormonal medications are still needed for in-depth exploration of this finding.

Surgical treatment is usually recommended for patients if the endometriosis is deep, causes severe pain or affects fertility. However, the correlation between the surgical management and the incidence of SLE is still uncertain. A case–control study focusing on European-Americans and African-Americans revealed a significantly increased rate of hysterectomy among patients with SLE^35^. However, another case–control study in the United States demonstrated the protective effect of prior hysterectomy (odds ratio 0.55; 95% CI 0.31–0.99)^36^. According to our results in Table 4, the non-surgical treatment group had the highest risk of SLE among the three subgroups (aHR 2.55; 95% CI 1.44–4.51). However, when focusing on the comparison of surgical treatment group and non-surgical treatment group, there was no statistical significance between their risks of SLE. Similar results were presented in another Taiwanese cohort study, showing the limited influence over the risk of SLE for endometriosis patients under surgical treatment^37^. It is worth noticing that owing to the lack of information on the severity of patients' endometriosis in our dataset, we were unable to adjust for this variable. More large-scale, carefully designed studies in the future are needed to clarify the effect of surgical intervention on the risk of SLE.

Our study is noteworthy because we found that with the use of corticosteroids, female participants who had endometriosis did not have a significant risk of SLE (HR 1.45; 95% CI 0.76–2.77). In contrast, corticosteroids-free participants who had endometriosis were observed to have a significantly higher HR of SLE as 9.29 (95% CI 2.14–40.25). Similar study by Lin, et al. did not stratified patients with the use of glucocorticoids or NSAIDs^37^. In fact, corticosteroids have been commonly used to treat SLE due to their anti-inflammatory and immunosuppressive effects. Corticosteroids effectively ablate the inflammation by suppressing the activity of cyclooxygenase enzymes (COX-1 and COX-2) and downstream proinflammatory factors such as prostaglandin and thromboxane^38^. Corticosteroids are also widely recognized for their immunosuppressive actions by repressing multiple immunomodulatory transcription factors. Previous researches revealed a long-term exogenous corticosteroids exposure may induce corticosteroid resistance, which will lead to increased susceptibility to autoimmune diseases^39^. However, the present study did not demonstrate similar results in patients with endometriosis. Our speculation to our results is that when received corticosteroids, the patient's autoimmune response was suppressed by corticosteroids, attenuating the additional risk of SLE for endometriosis patients. The association was not observed in participants using NSAIDs. This can be explained since corticosteroids not only have a stronger anti-inflammatory effect than NSAIDs, but also act as immunosuppressive agents. Further understanding about the pathogenesis of SLE and more careful studies on corticosteroids are necessary to confirm our speculation.

It needs to be further explained that drugs are classified into prescription drug, indicator drug and over-the-counter (OTC) drug in Taiwan. The latter two can be purchased without prescriptions from physicians, which means that our participants may purchase some specific types of drug such as hormone or contraceptives or OTC NSAIDs from the pharmacies outside the record of our database. Except for few types of oral gel and topical ointments, corticosteroids are all prescription drugs. As a result, the lack of complete records on participants' use of NSAIDs, hormones treating for endometriosis, glucocorticoids may also confound the results. However, the coverage of the NIH programme is 99% of the population and patients in Taiwan can get easy access to medical care could reduce the cofounding.

The limitations of our research should be highlighted. First, we were unable to acquire laboratory data that could monitor participants’ immune systems or hormone levels to further validate our hypothesis as this is a retrospective cohort registration study. Second, some risk factors, such as smoking, alcohol consumption, body mass index, and family history were not recorded in our database^40^. These variables may confound our discovered associations between endometriosis and SLE to some degree. Besides, both SLE and endometriosis are diseases that are often delayed in diagnosis due to their atypical onset symptoms. Also, they both share similar age-specific incidence. As a result, even patients diagnosed with SLE before index date were all excluded, it is still possible that a small number of patients with undiagnosed SLE were enrolled in the cohorts, which could influence the association. In addition, certain subgroup analyses may be limited by the small number of cases, which may increase the chance of erroneously obtaining subgroup effects and interactions. Moreover, given the racial differences in the incidence of systemic lupus erythematosus, and the lack of diversity in ethnicities in Taiwan population, the results of this study may not be generalized for other races/ethnicities. Finally, patients in the endometriosis group might have had a greater number of medical visits and therefore a greater chance of being diagnosed with SLE, which would represent detection bias.

Our research possesses several major advantages. First, as mentioned above, the use of data from large-scale, nationwide, randomly assigned samples from the LHID/NHIRD could reduce selection bias. Second, all claim data in LHID/NHIRD was carefully reviewed by experts and clinicians to ensure validity. Finally, although SLE has a gradual onset and is commonly underdiagnosed for years, a 12-year follow-up in our study would be sufficient to minimize underestimation of the incidence of SLE.

In conclusion, we report a significant association between endometriosis and SLE. However, further basic medical researches are needed to clarify the link and mechanisms between endometriosis and SLE.

## Supplementary Information

Supplementary Information.

## Data Availability

All data relevant to the study are available from the National Health Insurance Research Database (NHIRD), which is provided by the National Health Insurance (NHI) administration, Ministry of Health and Welfare of Taiwan and the National Health Research Institutes (NHRI) of Taiwan. It is not publicly available because it restricts only the researchers or clinicians who applied and signed an agreement with NHRI are eligible to apply for the National Health Insurance Research Database (NHIRD). The following is the official website of the NHIRD (https://nhird.nhri.org.tw/).
