# Supplementary material for: Association between endometriosis and risk of systemic lupus erythematosus
Source: Sci Rep. 2021 Jan 12;11:532. doi: 10.1038/s41598-020-79954-z (PMC7803765; doi:10.1038/s41598-020-79954-z)
Supplement: Supplementary file 1 — Supplementary Information. [file 41598_2020_79954_MOESM1_ESM.docx]

**Supplementary Information**

**Association Between Endometriosis and Risk of Systemic Lupus Erythematosus**Yu-Hsi Fan, Pui-Ying Leong, Jeng-Yuan Chiou, Yu-Hsun Wang, Ming-Hsiang Ku, James Cheng-Chung Wei

**Supplementary Table S1.** Lists of surgeries as surgical treatment for endometriosis

| **Laparoscopic surgery** | |
| --- | --- |
| **Read Code** | **Read Term** |
| 80014C | Laparoscopic fulguration or excision of pelvic endometriosis — Minimal to mild |
| 80029C | Laparoscopic fulguration or excision of pelvic endometriosis — Moderate |
| 80031C | Laparoscopic fulguration or excision of pelvic endometriosis — Severe |
| 80416B | Laparoscopy hysterectomy |
| 80807C | Laparoscopic partial or complete adnexectomy - unilateral |
| 80812C | Laparoscopic partial or complete adnexectomy - bilateral |
| **Hysterectomy** | |
| **Read Code** | **Read Term** |
| 80404C | Subtotal hysterectomy |
| 80412B | Extended hysterectomy |
| 80414B | Hysterectomy vaginal radical,Schauta type procedure |
| 80421B | Complicated total hysterectomy |
| 81005C | Subtotal hysterectomy after Cesarean section |
| 81029C | Total hysterectomy after Cesarean section |
| **Adnexectomy** | |
| **Read Code** | **Read Term** |
| 80802C | Partial or complete adnexectomy - unilateral |
| 80811C | Partial or complete adnexectomy - bilateral |

| Endometriosis | N | Crude HR | 95% CI | Adjusted HR | 95% CI |
| --- | --- | --- | --- | --- | --- |
| No | 16758 | 1 |  | 1 |  |
| Endometriosis with laparoscopic diagnosis | 525 | 3.87 | 0.90**–**16.70 | **4.74** | **1.07–20.93** |

**Supplementary Table S2.** Sensitivity analysis for HRs and 95% CIs of SLE by limiting the endometriosis diagnostic criteria to laparoscopic-diagnosed endometriosis

Bold font represents statistical significance (P < 0.05). An adjusted hazard ratio (HR) is calculated from crude HR after adjusting for age, hypertension, chronic liver disease, corticosteroids use, NSAIDs use, and hormonal medications use. **Abbreviation:** SLE, systemic lupus erythematosus; HR, hazard ratio; CI, confidence interval.
